# Evaluation of Plant Stress Monitoring Capabilities Using a Portable Spectrometer and Blue-Red Grow Light

**DOI:** 10.3390/s22093411

**Published:** 2022-04-29

**Authors:** Trina Merrick, Ralf Bennartz, Maria Luisa S. P. Jorge, Stephanie Pau, John Rausch

**Affiliations:** 1 Naval Research Laboratory, Remote Sensing Division, 4555 Overlook Ave. SW, Washington, DC 20375, USA; 2 Department of Earth and Environmental Science, Vanderbilt University, 5726 Stevenson Center, Nashville, TN 37240, USA; ralf.bennartz@vanderbilt.edu (R.B.); malu.jorge@vanderbilt.edu (M.L.S.P.J.); john.rausch@vanderbilt.edu (J.R.); 3 Space Science and Engineering Center, University of Wisconsin—Madison, 1225 W Dayton St., Madison, WI 53706, USA; 4 Department of Geography, Florida State University, 113 Collegiate Loop, Tallahassee, FL 32306, USA; spau@fsu.edu

**Keywords:** spectroscopy, chlorophyll fluorescence, vegetation indices, NDVI, PRI, photosynthesis, photosystem inhibition

## Abstract

Remote sensing offers a non-destructive method to detect plant physiological response to the environment by measuring chlorophyll fluorescence (CF). Most methods to estimate CF require relatively complex retrieval, spectral fitting, or modelling methods. An investigation was undertaken to evaluate measurements of CF using a relatively straightforward technique to detect and monitor plant stress with a spectroradiometer and blue-red light emitting diode (LED). CF spectral response of tomato plants treated with a photosystem inhibitor were assessed and compared to traditional reflectance-based indices: normalized difference vegetation index (NDVI) and photochemical reflectance index (PRI). The blue-red LEDs provided input irradiance and a “window” in the CF emission range of plants (~650 to 850 nm) sufficient to capture distinctive “two-peak” spectra and to distinguish plant health from day to day of the experiment, while within day differences were noisy. CF-based metrics calculated from CF spectra clearly captured signs of vegetation stress earlier than reflectance-based indices and by visual inspection. This CF monitoring technique is a flexible and scalable option for collecting plant function data, especially for indicating early signs of stress. The technique can be applied to a single plant or larger canopies using LED in dark conditions by an individual, or a manned or unmanned vehicle for agricultural or military purposes.

## 1. Introduction

Spectrometer measurements of chlorophyll fluorescence (CF) capture photosynthetic activity and plant function information, have been shown related to gross primary production (GPP) and carbon uptake, and can capture signals of plant stress, e.g., [1,2,3]. CF is a physiological process undergone by plants to dissipate excess energy from photosynthesis to protect tissues by emitting radiation mostly in the red and far-red region of the spectrum (approximately 650 to 850 nm) and, as a by-product of photosynthesis, provides a mechanistic link to plant function [4,5]. Thus, CF offers opportunities to assess vegetation status at leaf, plant, canopy, ecosystem, and global levels across spatial and temporal scales, e.g., [6,7,8,9,10,11,12,13,14,15,16,17,18,19,20,21,22,23].

Two predominant categories of CF measurement techniques are active and passive techniques. Widely used active techniques employ lasers, light emitting diode (LED) illumination, or lamps to excite chlorophyll and instruments, such as cameras or spectrometers, to record CF promptly or delayed and include techniques, such as pulse-amplitude-modulation (PAM) [4,7,12,16,24,25,26,27,28,29,30,31,32,33,34,35]. By activating photosynthesis with a light of a known, narrow wavelength, the PAM technique allows measurements of several aspects of photosynthetic activity in ambient light conditions in great detail. Such active techniques, however, are restricted mainly to leaf level and have intricate protocols to follow for experiment design and are not easily scaled up. The other category is measuring CF in ambient conditions, mostly in sunlight. In contrast to PAM, CF measured with the so-called passive techniques can be made at leaf to ecosystem scales using spectrometers mounted on the laboratory bench, towers, drones, or on satellites. In addition to allowing data collection at multiple scales, passive CF measurements have also been shown to scale effectively to larger levels, such as globally, and are more generalizable because the light activating photosynthesis is usually sunlight, unlike the active technique [23,28,36,37,38,39,40,41].

CF can be challenging to measure because the CF signal is small compared to the amount of sunlight, or potentially alternative light, the plant requires to drive photosynthesis. For this reason, most methods to examine CF have remained complex, time intensive, require extensive knowledge of the instrument and illumination source specifications, uncertainties, protocols, and results are difficult to interpret. For example, current passive CF measurement methods include retrievals under sunlight requiring one of several algorithms to exploit the oxygen absorption bands that coincide with the CF emission spectrum centered at approximately 687 and 761 nm. The retrieved CF values are used for estimates or as model inputs to estimate the CF spectra, e.g., [22,42,43,44,45,46]. Another option is to use an actinic light source having a “window” coincident with the CF spectrum can allow CF to be captured with a spectroradiometer. In many cases, a filter is placed within a chamber, such as within the source chamber for a leaf clip, to block incoming light in the CF wavelength range and then record the plant CF spectra in that “window”. This filtering and measurement process can be tedious, require expensive components, and, in the case of using the leaf clip, restrict measurements to leaf scale similar to the PAM method [42,47,48,49,50]. Therefore, despite advantages over active techniques, passive measurements of CF have remained complex.

With the goal of identifying a potentially more straightforward CF-based technique to detect plant stress, an initial investigation was carried out to address the following: (1) what level of sensitivity to plant stress can be measured using a spectrometer and blue-red light emitting diode grow light, and (2) are CF spectra or CF metrics derived from these spectra indicative of stress earlier than visual inspection or traditional vegetation indices? Specifically, an Analytical Spectral Devices (ASD)/Malvern FieldSpec^®^ HandHeld 2™ Spectroradiometer (HH2) with a blue-red grow light emitting diode lamp (MiracleLED^™^ 2.2 Watt; LED) was used to make repeated measurements of a target plant treated with a photosystem inhibiting poison. The ability to detect CF, the sensitivity of the CF region of the spectra, and metrics derived from the CF region were tested and metrics were compared to measurements of the normalized vegetation index (NDVI) and the photochemical reflectance index (PRI), which are often used to assess vegetation function.

## 2. Materials and Methods

**Figure 1:**
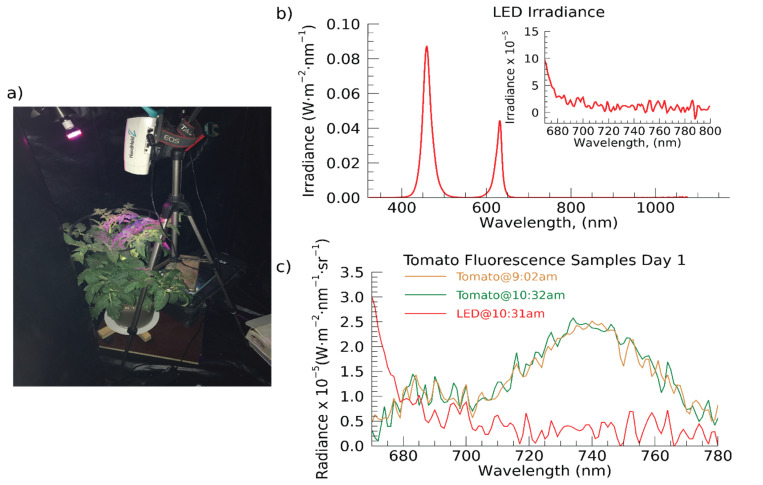
(**a**) The experimental instrument setup included the handheld ASD/Malvern HandHeld-2 Pro spectroradiometer (Boulder, CO, USA; HH2) and a blue-red LED grow light. A full spectrum lamp is added here for the purposes of a clear photo only. (**b**) Plot of blue-red light emitting diode grow light (MiracleLED™ 2.2 Watt; LED) spectrum, measured by recording the spectrum of the blue-red LED incident on a Spectralon^®^ white reference panel (Boulder, CO, USA; WR). Inset: zoom in to a portion of the fluorescence range from 670–800 nm. (**c**) Two sample spectra of the tomato plant within the fluorescence range (approximately 670 nm–780 nm with the spectrum of the WR measurement of the LED for reference.

Healthy, similar-sized tomato plants (*Solanum lycopersicum*), chosen for applicability for follow up studies, were placed in two chambers (target and control) of a dark tent in the laboratory (Figure 1a). On Day 1 of the experiment, a dose of a photosystem II (PSII) inhibitor, an algicide/herbicide called 3-(3′,4′-dichlorophenyl)-1,1-dimethylurea (DCMU) mixed with 300 mL water was applied to the soil of the target plant. DCMU blocks the electron flow from photosystem II (PSII) during photosynthesis, thus inducing stress when plants cannot effectively convert incoming energy to useful forms for carrying out proper functioning. For this reason, DCMU makes an efficient weed killer, for instance. The large application amount relative to what might be used for weed control in a natural setting was chosen to illicit an intentional stress response due to photosynthetic system shutdown in order to test the capability to detect and track [51]. In this scenario, CF would be expected to rise dramatically in the beginning stages because the energy supplied by incoming illumination would be blocked from entering PSII and a greater amount of excess energy would be given off as CF. After this initial increase, a decline in function results in a decline in CF.

The HH2 was mounted on a standard tripod above the target tomato plant canopy in the dark tent enabling a field of view of approximately 29 cm diameter (Figure 1a). An LED was positioned in each chamber using flexible bulb holders from a single lamp base to provide incoming photosynthetically active radiation (PAR; ~400–680 nm), one illuminating the target tomato plant and one illuminating the control. The LED emits light only in the blue and red regions, leaving a “window” in the upper red and far-red regions where plants emit CF as a by-product of photosynthesis (Figure 1b). Using a timer, the LEDs were turned on at 7:00 a.m. CDST each day and turned off at 7:00 p.m. CDST each evening, during which the HH2 was set to record a spectral measurement every 30 min. Figure 1c shows examples CF spectra on Day 1 of the experiment and the typical two-peak feature of a CF spectrum are distinguishable. For reference, an incoming (LED) spectrum (measured using a Spectralon^®^ white reference panel (WR)) is overplotted to illustrate the CF and LED spectra are clearly distinguishable from one another. However, it should be noted that the magnitude of the LED spectrum is lower when illuminating the plant than when illuminating the WR (albedo of ~1, while the plant albedo ~0.4).

Once daily, a Spectralon^®^ white reference panel (WR) was placed above the target plant canopy and an irradiance measurement of the LED was made. In addition, a measurement of the target and control plant and the WR were made under an ASD/Malvern Illuminator Reflectance Lamp (IRL, 70 W stable quartz-tungsten-halogen calibrated light source, ASD/Malvern, Boulder, CO, USA). The spectra under IRL were used to calculate daily normalized difference vegetation index (NDVI) and photochemical reflectance index (PRI) measurements. The daily reflectance of the control plant did not change throughout the experiment (not shown).

The CF portion (650–800 nm region) was extracted from each observation made between 8:00 a.m. CDST and 6:00 p.m. CDST. The first and last 1–2 measurements of the day were omitted to avoid erroneous measurements due to mismatch of the lamp timer and HH2 internal clock/auto-timer setting or any drift in either timer. A daily average CF spectrum and standard deviation at each wavelength was calculated. The resulting CF spectra were smoothed (central-moving average, window = 5). In addition to the full CF spectrum, CF values at 685 nm and 740 nm were extracted (F_red_, F_far_, respectively) and then the F_red_/F_far_ ratio calculated [21,51,52,53].

Daily NDVI [54] was calculated by calculating reflectance from the target plant spectrum and the IRL reference spectrum taken once each day and using:

(1) $$\mathrm{NDVI}=\frac{R_{800}-R_{680}}{R_{800}+R_{680}}$$

and PRI [55] as

(2) $$\mathrm{PRI}=\frac{R_{531}-R_{570}}{R_{531}+R_{570}}$$

where $R_{\lambda }$ is the reflectance and λ is the indicated wavelength in nm. To examine the relative capabilities of the measurements to capture the dynamics of photosynthetic function, we also calculated the proportion of maximum NDVI, PRI, F_red_, and F_far_ as a time series.

Custom programs to read, write, and batch process the spectra from their proprietary format as well as specifically process and make figures from the experiment were written in Interactive Data Language (IDL; L3Harris Geospatial, Boulder, CO, USA).

## 3. Results

**Figure 2:**
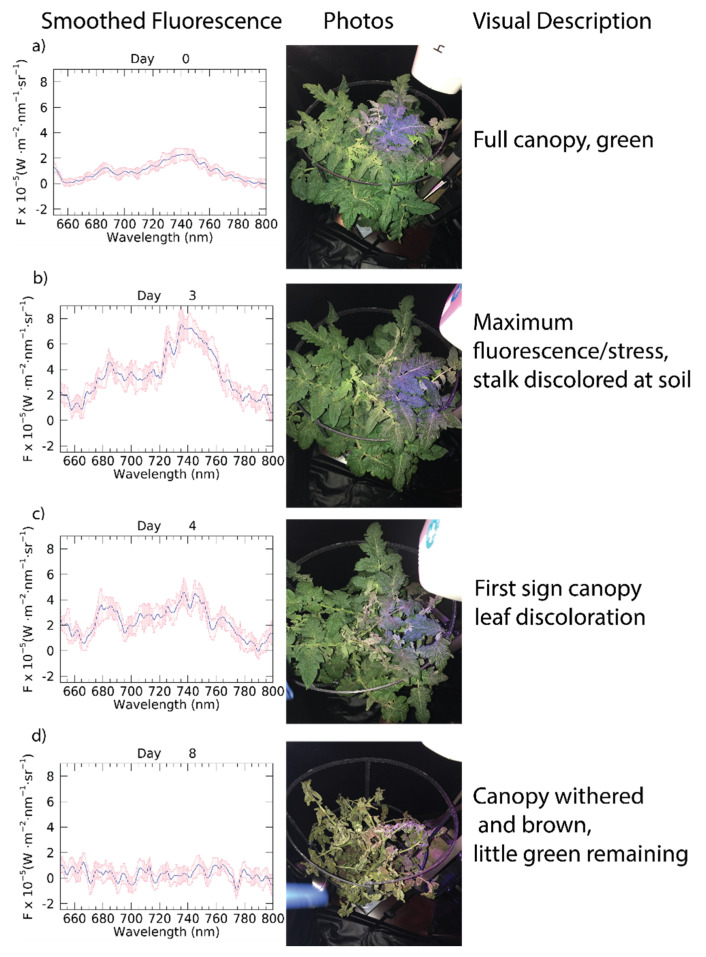
CF spectra, images and descriptions of selected days of experiment: (**a**) Day 0, (**b**) Day 3, (**c**) Day 4, (**d**) Day 8. Left column: Daily average smoothed fluorescence (F_sm_), blue lines indicate F_sm_ spectra, shaded pink region indicates standard deviation for the day. Middle column: photographs of tomato plant each day. Right column: description of visual inspection of plant for each day. Daily fluorescence spectra, pictures and visual descriptions are included in Appendix A.

**Figure A1:**
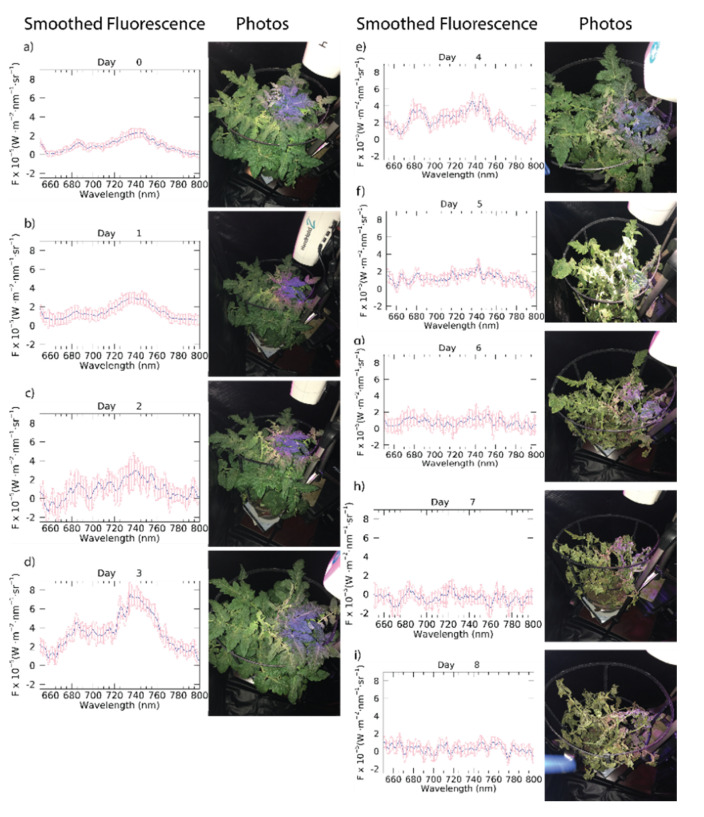
Daily average smoothed fluorescence and photographs of target tomato plant for all days of experiment (Days 0 to 8 spectra and photographs labelled (**a**–**i**), respectively).

Days 0 and 1 show the near and far-red peaks expected for the fluorescence range, which become less distinguishable on Days 2–4 as the variability for each increase (Figure 2a and Figure A1a–e). While the plant canopy is visibly unchanged from Day 0 to Day 3 (Figure 2a and Figure A1a–d), the red peak increases by Day 3 compared to Day 0 by approximately 4 times, while the far-red peak increases by 3.5 times, likely due to the increase in CF corresponding to photosynthetic inhibition. After the Day 3 peak in the fluorescence values, both peaks are decreased by Day 4, when the first signs of leaf discoloration and some wilting appear in the canopy, yet CF is still elevated compared to before DCMU application (Figure 2c and Figure A1e). By Day 5 of the experiment, the two peaks of fluorescence are indistinguishable, eventually reducing to noise around zero by Day 7 (Figure A1f–h) and most leaves on Day 5 show some withering, discoloration, spots, or slightly less green at a minimum and some portions of soil began to show through the canopy when viewed from above continuing to degrade through Day 8, the final day of measurements, the recorded CF spectra and photos show little to no green vegetation being measured by the HH2 (Figure 2d and Figure A1a–i).

**Figure 3:**
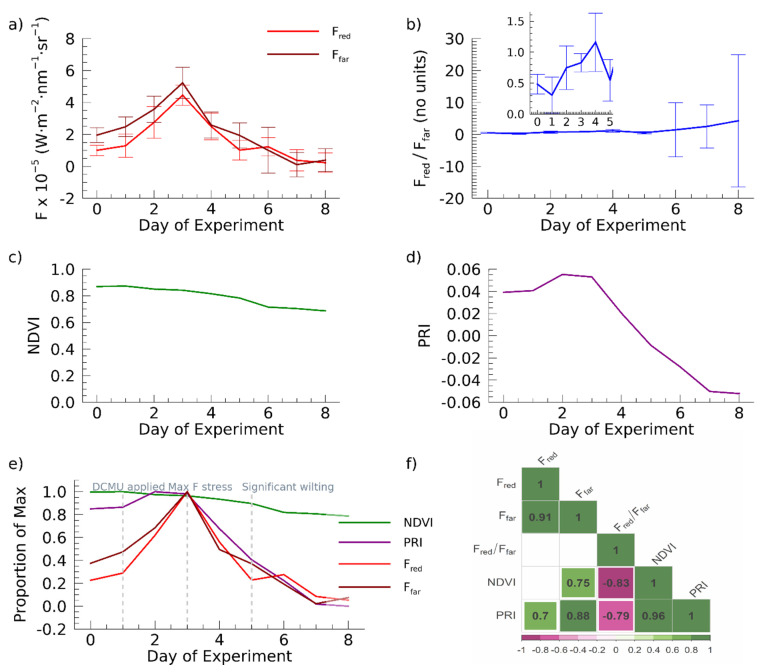
Results of plant stress experiments. CF metrics, NDVI and PRI measurement time series for the experiment. (**a**) Daily mean_,_ F_red_ and F_far_, (**b**) Daily mean F_red_/F_far_, Inset plot of the first five days of the experiment highlighting the trend of increasing F_red_/F_far_, (**c**) The normalized difference vegetation index (NDVI) taken one time daily for the experiment. (**d**) The photochemical reflectance index taken one time daily for the experiment (**e**) F_red_, F_far_, NDVI, and PRI plotted as the proportion of maximum over the course of the experiment. (**f**) Pearson Correlations among the mean values. Only significant correlations (*p*-values < 0.05) are shown along the lower right half. Blank areas in the lower right half indicate insignificant correlations (*p*-values > 0.05). Error bars indicate the standard deviation for each day.

From Day 0–3, F_red_ and F_far_ increase dramatically (333% and 166%, respectively, Figure 3a) going from approximately 0.2 and 0.3 of maximum to 1.0 (maximum values) in this time period, while PRI increased from 0.8 of maximum to 1.0, an increase of 35%, only subtly capturing photosynthetic response (Figure 3d,e). On Days 3–5, F_red_ and F_far_ decline close to Day 2 levels (183% and 32% of Day 0 values, respectively) reaching approximately 0.2 and 0.3 of maximum (Figure 3a,e) and PRI performs more similarly going from maximum (reached on Day 2) to 0.4 of maximum, indicating the pigment changes occurring in the plant leaves. Day 5 and beyond F_red_ and F_far_ are below the Day 0 values for F_red_ and F_far_, reaching values on Day 8 of 84% and 80% decrease from Day 0, while PRI reaches a value of −0.05 by Day 8 capturing pigment changes and structural changes ion with structural changes in the plant, e.g., [1,13] (Figure 3d,e).

F_red_/F_far_ increases to a maximum value of 1.2 on Day 4, when F_red_ surpasses the value of F_far_, then declines (Figure 3b inset shows Day 0–Day 5 detail) which highlights the capability of the instrument to record CF signals that increase when PSII is initially blocked and excess energy dissipation as CF increases, capturing initial stress reactions. The F_red_/F_far_ (Figure 3b) shows variability so large after Day 5 (due to increasingly low values of F_red_ and F_far_ compared to instrument noise) that the trend over the first five days is obscured by the scale of the graph. The decrease in signal for the F_far_ region of the spectrum results in dramatic increase in noise and extreme variability in F_red_/F_far_ on Days 6–8. 

In contrast, NDVI decreases throughout the experiment from approximately 0.87 to 0.69 at an average of 0.03 per day (a total decrease of only 21%, and approximately 0.8 of maximum value) and does not capture dynamic changes during photosystem inhibition and does not show a response to the DCMU poisoning of the plant prior to visible signs of stress on the plant on Day 4 (Figure 3c,e). Additionally, the value of 0.67 for NDVI at the end of the experiment indicates NDVI does not fully capture the reduced functioning of the plant (Figure 3c).

A comparison of the daily NDVI, PRI, F_red_, and F_far_ using the proportion of maximum of each (Figure 3e) highlights the relative degree to which these metrics capture changes in the response of the plants to stress. In this manner we show the large changes in PRI, F_red_, and F_far_ as compared to the change in NDVI. Figure 3e also illustrates the difference in timing of the maximum values, which is the beginning of the experiment for NDVI, Day 2 for PRI, and Day 3 for F_red_ and F_far_, further supporting that NDVI does not respond clearly to the initial stress, PRI has a potential moderate response, but F_red_ and F_far_ capture both the increase in dissipated energy during the subsequent onset of stress and the decline in function. Pearson correlations (*r* values) among the metrics are shown in Figure 3f for comparison, especially between the CF metrics and RIs (NDVI and PRI). No significant correlation exists between F_red_ and NDVI, F_red_ and F_red_/F_far_, as well as F_far_ and F_red_/F_far_. However, a positive relationship exists between PRI and F_far_ (*r* = 0.88, *p* < 0.01) for PRI and F_red_ (*r* = 0.7, *p* < 0.05) as well as for NDVI and F_far_ (*r* = 0.75, *p* < 0.05). The F_red_/F_far_ is negatively correlated with both NDVI (*r* = −0.83, *p* < 0.01) and PRI (*r* = −0.79, *p* < 0.05). F_red_ and F_far_ are highly positively correlated and are likely autocorrelated which mathematically explains the lack of relationship to their ratio (F_red_/F_far_). PRI and NDVI are also strongly positively correlated (*r* = 0.96, *p* < 0.01).

## 4. Discussion

An overarching goal of this study was to confirm that CF at canopy level could be detected and monitored with a relatively straightforward measurement technique. The general expectation that the increase in CF emission that would coincide with photosystem inhibition would manifest in an increase in the far-red peak of the spectra was confirmed. While individual measurements were too noisy to discriminate half-hourly or hourly decreases in plant function, likely due to the combination of an uncalibrated illumination source (LED) and dynamic and complex response of fluorescence, it was found that smoothed signals on a daily basis yielded CF information that could be used for daily plant function information, especially for indication of stress. Based on the results, CF measurements with this technique promise to evaluate more precisely the function and the stress than NDVI and PRI, which are both reflectance-based indices (RI). CF spectra and CF metrics, especially F_red_ and F_far_, capture the initial photosynthesis stress response and the decline of plant function to a greater degree than PRI and NDVI highlighting the capability of CF to better monitor dynamics of plant status.

Because CF originates from photosynthetic machinery of plants, it was anticipated that if distinct CF signals were captured and distinguishable, the CF spectra and CF metrics would detect stress earlier and be more sensitive in monitoring it than RIs. NDVI is the most used vegetation index and is often referred to as a “greenness index” due to its widespread use to track large scale seasonality. NDVI is calculated from reflectance in the red and near-infrared portion of the spectrum, is sensitive to changes in biomass and leaf area index (LAI), is a good indicator of absorbed photosynthetically active radiation (APAR) of a canopy and mainly captures structural changes (biomass and LAI) seasonally [1,13,55]. However, the structural changes in the target plant lagged behind the functional response, i.e., conversion of short wavelength energy from the PAR range to longer wavelength emission byproduct of the photosystem in the CF range.

PRI is significantly positively correlated with both F_red_ and F_far_, while NDVI is only significantly correlated with F_far_. While NDVI and PRI can explain a good deal of variation in CF, RI sensitivity to plant function is lower than measurements of CF. PRI is also an RI, but unlike NDVI, has been shown to detect changes in photosynthetic activity on two timescales: (1) diurnally capturing responses of the xanthophyll cycle to changing illumination and (2) pH of thylakoid lumen and seasonally capturing changes in the chlorophyll-carotenoid ratios, i.e., pigment content of leaves. PRI has also been shown to correlate with light-use efficiency (LUE) of some vegetation [1,13,56,57,58,59]. In this experiment, CF response was more sensitive to the plant stress than either NDVI or PRI, but the decline in PRI over the last days of the experiment showed the changes in leaf pigment present were detectible with the PRI and was more sensitive to changes in the canopy than NDVI. This is consistent with several studies showing PRI as a good indicator of plant function, albeit the precise responses are still being studied for temporal and spatial interpretation in some ecosystems, e.g., [13,59].

The comparison of the portions of the CF spectrum, F_red_ and F_far_, and their ratio, F_red_/F_far_, revealed dynamic responses to plant stress and show promise to make distinctions regarding physiology of the target plant. The shape of CF peaks for a plant at room temperature depend on leaf chlorophyll-a concentration, structure, and constituents, and the optical properties of the leaf determine the penetration depth of incident light and remission of CF from these depths [7,21,51,60,61,62,63,64]. As was the case in this investigation, studies show the F_red_ region suffers from reabsorption of CF photons to a greater degree than the F_far_ region, explaining the lower peak of F_red_ than F_far_, e.g., [3,65]. Additionally, F_red_ increased at a relatively higher rate than F_far_ in response to DCMU poisoning, which was expected from this particular herbicide. DCMU as a photosystem inhibitor most affects PSII; thus, it is expected that the PSII inhibition of photosynthesis would manifest as a dramatic increase in F_red_, which is the case in this study [28,62,66]. While there is a PSI and PSII contribution to both F_red_ and F_far_, F_red_ is produced dominantly by PSII and is more variable compared to F_far_. Furthermore, in healthy leaves, F_red_ is close to or less than F_far_, but in stressed leaves, F_red_ increases and F_far_ decreases [5,60,67]. It is for the reasons mentioned that the red peak (F_red_) and far-red peak (F_far_) and the red-far-red ratio (F_red_/F_far_) are metrics that have been utilized to detect stressed vegetation and estimate plant functioning status, and all three were indicative of changing function of the target plant in this experiment [53,62,68,69,70].

In this study, we demonstrate that a system using the HH2 and blue-red LED grow lamp to detect and monitor measure plant canopy level function, especially in the context of stress, has potential in a variety of applications. The capability to capture distinct signals of plant canopy stress earlier than traditional reflectance-based indices, NDVI and PRI, plus the capability to scale from small to larger canopies holds promise for both research and applications in the lab or field, at night for instance. Recently, two larger scale studies have employed LEDs with spectroscopy at night to study fluorescence responses, one in a forested area examined steady state responses of canopy and understory in a scots pine forest [71] and another used tractor mounted spectrometer and LEDs to measure fluorescence and compare to aerial net primary production among varieties of soybean, both rainfed and irrigated [72]. In the study of soybeans, the authors also show that the F_red_/F_far_ revealed differences in plant function among cultivars, while traditional RI’s did not. Our results support findings in these studies, albeit on a smaller scale, such that might be found in a laboratory or greenhouse. Taken together, the results of this study show the potential of the HH2 sensor to provide robust plant function information with this technique.

## 5. Conclusions

This work presents a process to measure CF using relatively straightforward methods and interpretation compared to other approaches aiming to capture fluorescence responses. Additionally, the technique can be applied to a single plant or larger canopies using LED, in dark conditions. The ability to collect data at leaf, plant, and canopy levels reliably and scale between these levels effectively with measurable uncertainties could also be applied, for instance, using manned or unmanned vehicles in agricultural or military applications. This scaling cannot be accomplished as easily or at all with other photosynthesis measurement techniques, such as pulse amplitude modulated fluorimetry (PAM). These procedures can be effectively employed as-is or with modifications in vegetation research and applications in multiple ways. For instance, plant function on multiple timescales could be further investigated by employing calibration for LEDs and examining timescales where signals would be statistically significantly different. In addition, CF measurements with our technique could be used in conjunction with PRI and NDVI measurements to inform plant studies along multiple timescales, i.e., CF is sensitive on the finer scale to track photosynthetic function and PRI on longer timescales to inform changes in leaf pigment, and add NDVI for seasonal structure changes. Therefore, the HH2 and LED system presented has broad appeal to multiple vegetation research areas.

## Data Availability

All data and code are freely available. Location and access provided upon request to the primary author.

## Appendix A

Daily fluorescence spectra, images and visual descriptions of experiment.
